# Nonlinear Optical Molecular Switches for Alkali Ion Identification

**DOI:** 10.3390/molecules190710574

**Published:** 2014-07-21

**Authors:** Aurélie Plaquet, Benoît Champagne, Frédéric Castet

**Affiliations:** 1 Laboratoire de Chimie Théorique, UCPTS, Université de Namur (UNamur), rue de Bruxelles 61, B-5000 Namur, Belgium; E-Mail: benoit.champagne@unamur.be; 2 Institut des Sciences Moléculaires (ISM), Université de Bordeaux, UMR 5255 CNRS, Cours de la Libération 351, F-33405 Talence Cedex, France; E-Mail: f.castet@ism.u-bordeaux1.fr

**Keywords:** nonlinear optics, molecular switches, alkali ion sensing, hyper-Rayleigh scattering, quantum chemical calculations, merocyanine/spiropyran

## Abstract

This work demonstrates by means of DFT and *ab initio* calculations that recognition of alkali cations can be achieved by probing the variations of the second-order nonlinear optical properties along the commutation process in spiropyran/merocyanine systems. Due to the ability of the merocyanine isomer to complex metal cations, the switching between the two forms is accompanied by large contrasts in the quadratic hyperpolarizability that strongly depend on the size of the cation in presence. Exploiting the nonlinear optical responses of molecular switches should therefore provide powerful analytical tools for detecting and identifying metal cations in solution.

## 1. Introduction

For a few years now, the design of molecular switches with large nonlinear optical (NLO) contrasts has been the focus of a lot of experimental and theoretical investigations [1,2,3,4]. These systems, characterized by their ability to alternate between two or more different chemical forms displaying contrasts in one of their NLO properties, are of particular interest in the development of new photonic technologies including frequency doublers, space communications or biomedical imaging [5,6]. The commutation can be triggered by using various external stimuli such as a variation of pH [7,8,9], temperature [10], light [11,12,13], redox potential [14,15,16], or solvent polarity. At the molecular scale, the property to optimize is usually the first hyperpolarizability (β), which is defined as the second-order response of the electric dipole moment (μ) to an external electric field, whereas a few studies have reported molecules with second hyperpolarizability (γ) contrasts [17]. A wide variety of NLO switches have been proposed, based e.g., on the indolino-oxazolidine [7,8,9,18,19], anil [11,20,21,22,23] or spiropyran [24] moieties, in which the amplitude of the β contrasts can be modulated by structural modifications and chemical functionalizations, *i.e.*, by varying the nature and length of the π-conjugated backbone, and/or the nature and position of donor and acceptor substituents.

**Scheme 1:**
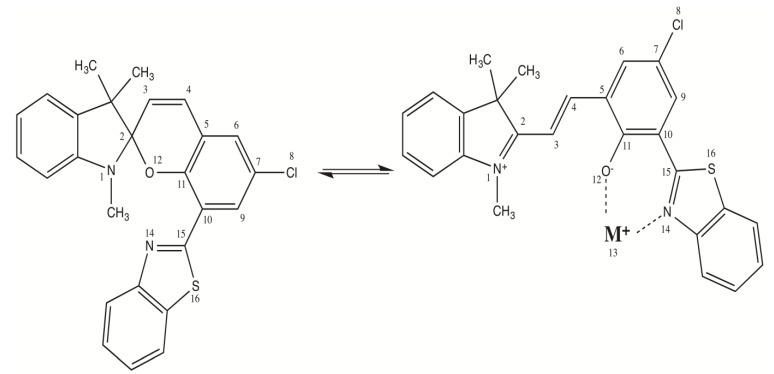
Equilibrium between the spiropyran form and the merocyanine form complexing an alkali ion.

Recently, we demonstrated by means of *ab initio* calculations that NLO switches based on the merocyanine/spiropyran equilibrium could also be exploited for selective identification of metal ions, thanks to the ability of the merocyanine form to complex cationic species such as alkali, alkaline earth, or transition metal ions. The design of molecular sensors able to recognize cations is of technological relevance with respect to many ecological, toxicological, or chemical issues [25,26]. In merocyanine/ spiropyran systems, the commutation processes were shown to induce large NLO contrasts that depend on the nature of the cation involved, providing therefore a powerful detection tool [27] complementary to standard techniques based on variations in the absorption or fluorescence spectra [28,29]. In this paper, we pursue our seminal investigation on a spiro[indoline-8-(benzothiazol-2-yl)-benzopyran] derivative (Scheme 1), whose metallochromic properties [30,31] and β contrasts [24,27] were previously reported, by analyzing its effectiveness to recognize alkali ions by means of variations in its linear and second-order nonlinear optical properties. In addition, we also concentrate on the thermodynamics of the cation complexation.

## 2. Results and Discussions

### 2.1. Molecular Structures and Thermodynamic Analysis

Representative bond lengths and torsion angles of the structures optimized in acetonitrile at the M06/6-311G*/Stuttgart1997 level are gathered in Table 1 for the spiropyran, the merocyanine, and the merocyanine complexing alkali ions. The largest variations appear for bonds 12–13 and 13–14 (ligand-metal, d_O-M_ and d_N-M_ distances) as well as 12–14 (claw opening distance), which evolves in parallel to the cation size, *i.e.*, Li^+^ < Na^+^ < K^+^ < Rb^+^ < Cs^+^. Note that the d_O-M_ distance is systematically shorter than the d_N-M_ one, due to the generally larger negative charge on the oxygen (q_O_ ϵ [−0.47, −0.54]) compared to the nitrogen (q_N_ ϵ [−0.41, −0.54]), where q_O_ has been obtained from Mulliken population analysis. The reverse trend is observed for the 11–12 and 14–15 bond lengths, which get slightly smaller (and the bond stronger) when the cation is larger, evidencing a weaker complexation effect. The cation complexation has also an impact on the bond length alternation [BLA = ½ (d_2-3_ + d_4-5_ − 2d_3-4_)] along the vinylic bridge linking the two aromatic moieties, which decreases from 0.039 Å for Li^+^ to 0.021 Å for Cs^+^. Indeed, a stronger complexation by the ligand induces the lengthening of the O-C bond, which then reduces the donor character of the ligand moiety (towards the accepting ring) and therefore increases the BLA. Owing to these π-conjugation effects and their modifications upon cation complexation, most bond lengths display systematic variations as a function of the cation size. Though the merocyanine moiety is planar, the θ_11-12-13-14_ dihedral angle increases significantly from Li^+^ (13.1°) to Rb^+^ (69.1°), which is related to the growing size of the cation and the need for a wider opening of the ligand claw.

**Table 1:** Bond lengths (Å), torsion angles (degrees), and bond length alternation ^a^ of the molecular structures optimized at the M06/6-311G*/Stuttgart1997 level. Solvent effects (CH_3_CN) are included via the IEFPCM scheme. See Scheme 1 for the atom labels.

	Spiro	Mero	Mero-Li^+^	Mero-Na^+^	Mero-K^+^	Mero-Rb^+^	Mero-Cs^+^
**1-2**	1.441	1.345	1.336	1.339	1.340	1.342	1.342
**2-3**	1.492	1.392	1.403	1.399	1.398	1.396	1.396
**3-4**	1.333	1.384	1.373	1.377	1.378	1.380	1.381
**4-5**	1.451	1.405	1.420	1.414	1.412	1.410	1.409
**7-8**	1.751	1.755	1.752	1.753	1.753	1.754	1.754
**11-12**	1.337	1.235	1.254	1.248	1.246	1.242	1.243
**12-13**	-	-	1.881	2.305	2.574	2.773	3.026
**13-14**	-	-	2.071	2.523	2.880	3.111	3.257
**12-14**	2.829	2.946	2.795	2.909	2.933	2.951	2.968
**10-15**	1.466	1.290	1.458	1.461	1.462	1.462	1.462
**14-15**	1.289	1.784	1.302	1.297	1.296	1.295	1.294
**15-16**	1.776	1.462	1.778	1.776	1.777	1.777	1.775
**θ_1-2-3-4_**	+135.7	+179.5	–179.7	–179.5	+179.2	–179.5	–179.2
**θ_2-3-4-5_**	+177.8	–179.7	–179.4	+178.2	–179.3	+179.9	+179.9
**θ_4-5-6-7_**	+177.9	–179.2	–179.5	+178.3	–178.9	+179.6	+179.8
**θ_11-12-13-14_**	-	-	+13.1	+44.5	+47.8	+52.7	+69.1
**BLA**	+0.139	+0.015	+0.039	+0.030	+0.027	+0.023	+0.022

^a^ BLA = ½ (d_2-3_ + d_4-5_ − 2 d_3-4_).

The global charge of the different fragments (S_i_) of the compounds were then computed at the M06/6-311G*/Stuttgart1997 level using the Mulliken approximation (Table 2). Upon complexing Cs^+^ the modification of the charge distribution is mostly located on the nearby ligands, of which the electron density increases. Then, going towards smaller cations, this charge transfer effect is enhanced and the ligands get more negative while the cations get less and less positive. As a matter of fact, the resulting excess positive charge is distributed over the rest of the system, and particularly on the two closer aromatic rings (S_5_ and S_9_) but also, to a lower extent, on the merocyanine part. Switching between the spiropyran and merocyanine forms is also characterized by charge reorganizations, resulting from the opening of the spiro function.

**Table 2:** IEFPCM/M06/6-311G*/Stuttgart1997 Mulliken charge distribution for the fragments (S_1_–S_11_) of the spiropyran, merocyanine, and merocyanine-M^+^. 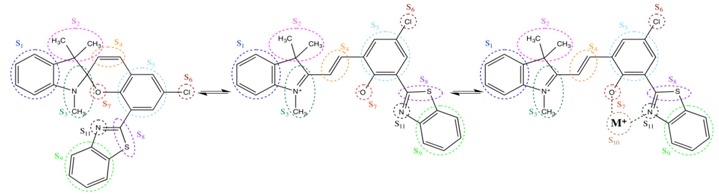

Fragments	Spiro	Mero	Mero-Li^+^	Mero-Na^+^	Mero-K^+^	Mero-Rb^+^	Mero-Cs^+^
**S_1_**	+0.240	+0.389	+0.426	+0.413	+0.410	+0.405	+0.404
**S_2_**	+0.006	–0.025	–0.011	–0.016	–0.016	–0.018	–0.019
**S_3_**	–0.100	+0.224	+0.257	+0.243	+0.243	+0.241	+0.240
**S_4_**	+0.106	–0.115	–0.053	–0.075	–0.082	–0.099	–0.106
**S_5_**	+0.259	+0.193	+0.296	+0.254	+0.241	+0.225	+0.197
**S_6_**	–0.084	–0.105	–0.094	–0.098	–0.098	–0.100	–0.100
**S_7_**	–0.382	–0.433	–0.538	–0.505	–0.500	–0.492	–0.470
**S_8_**	+0.310	+0.263	+0.406	+0.343	+0.322	+0.302	+0.302
**S_9_**	–0.009	–0.035	+0.072	+0.048	+0.029	+0.012	+0.011
**S_10_**	-	-	+0.783	+0.866	+0.886	+0.942	+0.954
**S_11_**	–0.346	–0.356	–0.544	–0.473	–0.435	–0.418	–0.413

Table 3 summarizes the main thermodynamic data for the complexation reaction of the alkali ion by the merocyanine form. The most exothermic reaction is the complexation of the largest cations (Rb^+^ and Cs^+^). The enthalpy of reaction evolves non-monotonically with the cation size, decreasing (in absolute value) from Li^+^ to K^+^, and then increasing to similar values for Rb^+^ and Cs^+^. To some extent, the Gibbs free energies behave similarly with the cation size, with however a more pronounced difference in the complexation energy of Li^+^ compared to that of the largest cations. This is attributed to the larger amplitude of the entropy in the case of Li^+^, due to the fact that the ligands are more tightly bound to the alkali atom. Indeed, the complexation reaction is accompanied by a decrease of the entropy and the amplitude of that reduction tends to increase as a function of the covalent character of the cation-ligand bonds.

**Table 3:** Enthalpy, entropy, and Gibbs free enthalpy of reaction (kcal mol^−1^ except the entropies in cal mol^−1^ K^−1^, at 298.15 K and 1 atm) for the complexation reaction calculated at the M06/6-311G* level in acetonitrile for different alkali cations.

Cations	ΔH^θ^	ΔS^θ^	ΔG^θ^
Li^+^	−15.5	−36.3	−4.8
Na^+^	−11.0	−31.7	−1.6
K^+^	−10.3	−26.4	−2.5
Rb^+^	−17.3	−28.1	−9.0
Cs^+^	−17.0	−25.9	−9.3

Selected vibrational frequencies calculated at the M06/6-311G* level in acetonitrile are listed in Table 4. Those around 1,600 wavenumber correspond to CO stretching vibrations and display a strong IR intensity. The frequency of these vibrational modes increases with the size of the cation. Indeed, smaller cations lead to a larger covalent character of the oxygen-metal bond, which translates into a smaller CO bond strength (as indicated by the increase of the CO bond length, see Table 1) and therefore into a smaller vibrational frequency. An opposite behavior is observed for the low frequency mode (684–495 cm^−1^), which presents a strong O-cation and N-cation stretching character. In this case, the smaller the cation, the stronger the covalent bonds and the stronger the bond strengths.

**Table 4:** Frequencies (cm^−1^) and IR intensities (km mol^−1^) of selected vibrational modes for the merocyanine-M^+^ systems calculated at the M06/6-311G* level in acetonitrile (IEF-PCM) for different alkali cations.

Cations	Frequency	IR Intensity
Li^+^	695.2	1
	1574.8	309
	1626.0	660
Na^+^	687.2	7
	1581.4	203
	1630.1	666
K^+^	686.7	7
	1584.2	205
	1631.6	615
Rb^+^	683.9	10
	1587.0	206
	1632.8	628
Cs^+^	684.5	9
	1586.5	194
	1632.0	589

### 2.2. Linear Optical Properties

The values of the transition energies, wavelengths, and oscillator strengths calculated at the TDDFT/ωB97X level with the 6-311G*/Stuttgart1997 basis sets are gathered in Table 5. Owing to the formation of a π-conjugated segment between the donor and acceptor moieties, the breaking of the spiro junction results in a decrease of the first excitation energy by 1.6 eV and a substantial increase of the oscillator strength. Complexing the big Cs^+^ atom leads to a small hypsochromic shift of the S_0_ → S_1_ transition (7 nm or 0.03 eV) whereas going towards smaller cations, this shift increases up to 18 nm (0.11 eV), consistently with the increase of the BLA along the vinylic linker (Table 1). Note that this hypsochromic shift goes in the opposite direction to what was observed and calculated for coumarin 343 fluoroionophores [32]. The effect of the cation size on the oscillator strengths is negligible.

**Table 5:** Wavelengths (λ_ge_, nm), transition energies (ΔE_ge_, eV), oscillator strengths (f_ge_) calculated at the TDDFT/ωB97X level with the 6-311G*/Stuttgart1997 basis sets for the different compounds. Solvent effects (CH_3_CN) are included via the IEF-PCM scheme.

	λ_ge_ (nm)	ΔE_ge_	f_ge_
Spiro	294	4.21	0.260
Mero	474	2.62	1.071
Mero-Li^+^	456	2.73	1.072
Mero-Na^+^	461	2.69	1.074
Mero-K^+^	463	2.68	1.066
Mero-Rb^+^	466	2.66	1.059
Mero-Cs^+^	467	2.65	1.059

### 2.3. Second-Order NLO Properties

Table 6 and Table 7 report the static and dynamic HRS first hyperpolarizabilities (β_HRS_), the depolarization ratios, as well as the β contrasts between the spiropyran and the merocyanine(-M^+^) calculated in acetonitrile using different levels of approximation, namely HF, ωB97X, and MP2. The dynamic (λ = 1064 nm) MP2 values were estimated using Equation (1).

The (static and dynamic) β_HRS_ values increase upon including electron correlation at the MP2 level, are considered as reference. Compared to HF values, this increase is of 141, 36, 147, 110, 95, 83, and 63% respectively for the spiropyran, merocyanine and the merocyanine complexing Li^+^, Na^+^, K^+^, Rb^+^, and Cs^+^. This results in variations of the first hyperpolarizability contrasts, in particular of the β_mero_/β_spiro_ contrast that decreases by about a factor of 2. When considering the ωB97X results, the electron correlation effects are about twice smaller than with the MP2 method. Except for the merocyanine where they lead to a decrease of β_HRS_ by 3%, the inclusion of electron correlation effects with the ωB97X functional enhances the HF β_HRS_ by 46%, 57%, 35%, 30%, 22%, and 16% for the spiropyran and the merocyanine complexing Li^+^, Na^+^, K^+^, Rb^+^, and Cs^+^, respectively. These exaltation effects are much amplified when considering the dynamic β_HRS_ values, as a consequence of the smaller ωB97X excitation energies that lead to stronger frequency dispersion.

More importantly, the MP2 calculations evidence a strong enhancement of the β_HRS_ response of the merocyanine form upon complexation, whose amplitude increases inversely to the size of the alkali ion. With the exception of Na^+^ and K^+^, which display similar NLO responses, the hyperpolarizabilities of the various merocyanine/cation complexes are significantly different, allowing unambiguous identification of the nature of the alkali metal. Similar trends are observed when using the ωB97X XC functional, whereas at the HF level the alkali complexation effects on β_HRS_ are much smaller and mainly go in the opposite direction. Besides, whatever the nature of the alkali, the complexation process does not induce remarkable variations in the values of the depolarization ratios (DR), which remain typical of one-dimensional π-conjugated systems dominated by a single diagonal β tensor component.

**Table 6:** Static (λ = ∞) and dynamic (λ = 1064 nm) HRS first hyperpolarizabilities, and depolarization ratios calculated at the HF, ωB97X, and MP2 levels with the 6-311+G*/Stuttgart1997 basis sets. Solvent effects (CH_3_CN) are included via the IEF-PCM scheme.

λ (nm)	Property	Spiro	Mero	Mero-Li^+^	Mero-Na^+^	Mero-K^+^	Mero-Rb^+^	Mero-Cs^+^
**HF**								
∞	**β_HRS_**	196	3766	3369	3659	3755	3774	3744
	**DR**	4.16	3.18	3.38	3.40	3.39	3.31	3.31
**1064**	**β_HRS_**	152	5568	5064	5523	5690	5654	5635
	**DR**	3.90	4.11	4.35	4.31	4.31	4.24	4.24
**ωB97X**								
∞	**β_HRS_**	287	3660	5297	4950	4866	4593	4353
	**DR**	4.48	3.02	3.95	3.77	3.69	3.91	3.55
**1064**	**β_HRS_**	240	11578	16234	15476	15294	14091	13847
	**DR**	5.22	4.64	4.89	4.87	4.86	4.81	4.80
**MP2**								
∞	**β_HRS_**	473	5133	8290	7667	7617	6899	6095 ^b^
	**DR**	4.56	3.17	4.55	4.01	4.15	3.92	3.74 ^b^
**1064**	**β_HRS_^a^**	367	7589	12461	11573	11542	10336	9173 ^b^
	**DR ^a^**	4.28	4.10	5.86	5.08	5.28	5.02	4.79 ^b^

^a^ Obtained with the multiplicative scheme, Equation (1); ^b^ Obtained with the 6-31G*/Stuttgart1997 basis sets.

**Table 7:** Static (λ = ∞) and dynamic (λ = 1064 nm) contrasts of HRS first hyperpolarizabilities of the spiropyran and the merocyanine(-M^+^) at the HF, ωB97X, and MP2 levels with the 6-311+G*/Stuttgart1997 basis sets. Solvent effects (CH_3_CN) are included via the IEF-PCM scheme.

Method	λ (nm)	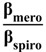	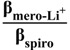	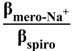	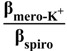	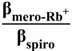	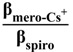
**HF**	∞	19.2	17.2	18.7	19.2	19.3	19.1
	**1064**	36.6	33.3	36.3	37.4	37.2	37.1
**ωB97X**	∞	12.8	18.5	17.2	17.0	16.0	15.2
	**1064**	48.2	67.6	64.5	63.7	58.7	57.7
**MP2**	∞	10.9	17.5	16.2	16.1	14.6	16.6 ^a^
	**1064**	20.7	34.0	31.5	31.4	28.2	32.2 ^a^

^a^ Obtained with the 6-31G*/Stuttgart1997 basis sets.

## 3. Computational Section

Geometry optimizations were performed at the density functional theory (DFT) level of approximation using the M06 exchange-correlation (XC) functional [33]. The 6-311G* basis set was used for all atoms except Rb and Cs, which were described by the Stuttgart 1997 basis set [34]. The components of the first hyperpolarizability tensor (β) were evaluated at different levels of approximation. The coupled-perturbed Hartree-Fock (CPHF) and the time-dependent Hartree-Fock (TDHF) approaches [35,36] were employed with the 6-311+G*/Stuttgart1997 basis sets to evaluate the static and dynamic (λ = 1064 nm) first hyperpolarizabilities, respectively. Secondly, in order to account for electron correlation effects, the static β components were computed by employing the second-order Møller-Plesset level (MP2) in combination with the finite field (FF) procedure [37] using the same basis sets. In these FF calculations, the higher-order contaminations were removed by adopting the usual Romberg procedure [38,39,40]. Then, the HF and MP2 results were combined in order to predict approximate dynamic MP2 values. This was achieved by using the multiplicative scheme [41,42,43], which consists in multiplying the static MP2 value by a corrective dispersion factor:

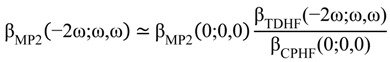
(1)


Additional calculations of the first hyperpolarizability were performed at the time-dependent density functional theory (TDDFT) level with the ωB97X XC functional [44]. This functional includes 15.77% and 84.23% of exact HF exchange at short- and long-range, respectively, and is among the best XC functionals to predict the excitation energies of push-pull π-conjugated systems. Among second-order NLO phenomena, we focused on the hyper-Rayleigh scattering (HRS) response, β_HRS_(–2ω;ω,ω) = β_HRS_ [45]. Assuming a non-polarized incident light propagating along the Y direction (in the laboratory frame), the intensity of the harmonic light scattered at 90° along the X direction and vertically (V) polarized (along the Z axis) is given by Bersohn’s expression [46]:

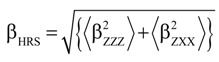
(2)


The depolarization ratio, DR, which reveals the shape of the NLO-phore, reads:

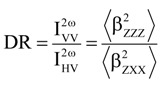
(3)
where 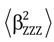 and 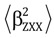 are orientational averages of β tensor components, which are proportional to the scattered signal intensities for vertically and horizontally-polarized incident signals, respectively. The orientational averages in Equations (2) and (3) were calculated without assuming Kleinman’s conditions, *i.e.*, without applying permutation symmetry rules on the Cartesian components of the hyperpolarizability tensor. All reported β values are given in atomic units [1 a.u. of β = 3.62 × 10^−42^ m^4^ V^−1^ = 3.2063 × 10^−53^ C^3^ m^3^ J^−2^ = 8.641 × 10^−33^ esu] within the T convention of [47].

The vertical excitation energies (∆E_ge_) and the corresponding wavelength λ_ge_ were calculated at the TDDFT level using the range-separated hybrid functional ωB97X and the combination of the 6-311G(d)/Stuttgart1997 basis sets.

Solvent effects on the linear and nonlinear optical properties were accounted by using the polarizable continuum model within the integral equation formalism (IEF-PCM) [48,49]. This model describes the molecular environment as a structureless polarizable continuum characterized by its macroscopic dielectric permittivity, *ε_ω_*, which depends on the frequency *ω* of the applied electric field. All the calculations were performed in acetonitrile (CH_3_CN: ε_0_ = 36.64, ε_∞_ = 1.806), using the Gaussian 09 package [50].

## 4. Conclusions

Structural, thermodynamical, vibrational, as well as linear and nonlinear optical properties of a spiro[indoline-8-(benzothiazol-2-yl)-benzopyran] derivative and of its merocyanine form have been evaluated by using first principles methods. In particular, calculations are used to unravel the variations of these properties that occur when the merocyanine form complexes alkali cations of increasing size, from Li^+^ to Cs^+^. The following trends have been observed, (i) the complexation of smaller cations leads to the formation of stronger metal-ligand bonds, larger geometrical relaxations, and larger charge redistributions over the whole system, that result in a stronger hypsochromic shift of the first excitation energy and in a larger enhancement of the first hyperpolarizability, (ii) these structural, electronic, vibrational, and optical properties are less impacted by the complexation of larger cations, and (iii) the enthalpies of complexation show a minimum for the Na^+^ and K^+^ cations for which the covalent bond is weaker than in the case of Li^+^, whereas the opening of the claw is too large to match the cation size as in the case of Rb^+^ and Cs^+^. The broad range of variation of the first hyperpolarizability as a function of the cation size confirms the potential of the second-order NLO responses of this merocyanine for detecting and identifying metal cation.
